# Access to preventive care by immigrant populations

**DOI:** 10.1186/1741-7015-10-55

**Published:** 2012-05-31

**Authors:** Mark Fort Harris

**Affiliations:** 1 Centre for Primary Health Care and Equity, University of New South Wales, Sydney, New South Wales, Australia

**Keywords:** Cardiovascular risk, immigrants, preventive care, primary care

## Abstract

Many immigrant populations lack access to primary health care. A recently published study on cholesterol screening among immigrant populations in the US found disparities in cholesterol screening in those originating from Mexico, largely due to limited access to healthcare. This inverse care affects immigrants in many destination countries despite their greater health need.

Please see related article: http://www.equityhealthj.com/content/11/1/22

## Background

Immigrant and refugee populations suffer a significant burden of disease, and health systems face significant challenges in addressing the health of migrant populations globally [1]. Despite this, there has been comparatively little research on inequities in access to preventive care experienced by immigrant and refugee populations.

Patterns of cardiovascular disease are complex in immigrant populations. 'Healthy migrant' selection effects have been observed among migrants in some countries, with associated reduced risk of disease [2]. Certainly, the cardiac risk among migrants in many migration destination countries is highly variable. However, where it is initially lower, their cardiovascular risk usually 'normalizes' to become the same as or worse than the rest of the population [3]. Stroke risk is less equivocal, tending to be consistently higher among immigrants. This is a pattern that is in part attributable to higher rates of hypertension and diabetes [4].

A study by Stimpson *et al*. [5] on cholesterol screening among immigrant populations was recently published in the *International Journal of Equity in Health Care*. This identifies persistent disparities in self-reported cholesterol screening for immigrants compared to non-immigrants including Hispanic populations. Using data from the 1998 to 2008 National Health and Nutrition Examination Surveys, the authors found 70.9% of immigrants originating from Mexico recalled being screened, compared with 80.1% of those born in the US and 77.8% of US-born Hispanic persons. This is consistent with other studies that have found higher rates of undiagnosed diabetes among US-Mexican border populations [6]. Both are examples of the inverse care law (in which those at greatest need receive less care) [7].

The disparities in cholesterol screening in Stimpson *et al.*'s study disappeared after adjusting for reduced healthcare access related to lack of health insurance among immigrants in the US. The non-insurance rates among people of Mexican origin in the US have been consistently high for over two decades [8]. This was attributable not only to their immigrant status but also the nature of their employment, which is linked to access to health insurance in the US. The importance of health insurance coverage as a determinant of disparities in immigrant access to healthcare has been reported in other studies [9].

Immigrants also have a lower probability of having a usual source of care [10]. This suggests that in addition to ensuring affordability, systematic policies are needed to better integrate immigrants into primary healthcare in advanced economies. Utilization of healthcare is of course not only influenced by affordability but also by patient factors such as health literacy. Immigrants are less likely to be aware of their cardiovascular risk or risk factors including cholesterol levels than the US-born population [11]. This lack of health literacy compounds the barriers of cost and affordability of preventive care for immigrant populations.

Therefore, primary healthcare policies need to address health literacy as well as language and cultural barriers to access by immigrant and refugee populations. This should involve access to interpreters (in person and by phone) as well as access to a range of translated materials including on the web. This needs to be complemented by training of health professionals in cultural competence, often overlooked in health professional education on refugee health, which is focused on infectious diseases and the psychological effects of trauma.

## Conclusions

All this has important implications for attempts to reform health policy in the US, where there is still no universal coverage of basic health insurance, and to revitalize primary care based on a model of the patient-centered medical home. Access to preventive care is particularly important among immigrant populations as it is a determinant of future risk of chronic disease, which in turn may lead to socioeconomic disadvantage. Unfortunately it is also very sensitive to cost disincentives. This problem is not unique to the US. Many countries, including Australia, restrict access by refugees to affordable medical care (which is available to rest of the population) until their asylum claims are accepted [12]. These highly vulnerable populations need more, not less, access to primary care.

## Competing interests

The author declares that they have no competing interests.

## Author Information

MFH is Executive Director, Centre for Primary Health Care and Equity, University of New South Wales. MFH is also a Volunteer Medical Practitioner, Sydney Asylum Seekers Centre.

## Pre-publication history

The pre-publication history for this paper can be accessed here:

http://www.biomedcentral.com/1741-7015/10/55/prepub
